# Preparation, structures and preliminary host–guest studies of fluorinated *syn*-bis-quinoxaline molecular tweezers

**DOI:** 10.3762/bjoc.6.39

**Published:** 2010-04-20

**Authors:** Markus Etzkorn, Jacob C Timmerman, Matthew D Brooker, Xin Yu, Michael Gerken

**Affiliations:** 1 Department of Chemistry, The University of North Carolina at Charlotte, 9201 University City Blvd., Charlotte, NC 28223, USA; 2 Department of Chemistry and Biochemistry, University of Lethbridge, Lethbridge, AB T1K 3M4, Canada

**Keywords:** crystal structure, fluorine, molecular tweezers, quinoxalines, self-association

## Abstract

A series of polycyclic frameworks with fluorinated *syn*-facial quinoxaline sidewalls has been prepared as potential molecular tweezers for electron-rich guest compounds. Our synthetic route to the cyclooctadiene-derived scaffolds **16a**–**d** takes advantage of the facile isolation of a novel spirocyclic precursor **9b** with the crucial *syn*-orientation of its two alkene moieties. The crystal structure of **16c** displays two features typical of a molecular tweezer: inclusion of a solvent molecule in the molecular cleft and self-association of the self-complementary scaffolds. Furthermore, host–guest NMR studies of compound **16c** in solution show chemical exchange between the unbound and bound electron-rich guest, *N*,*N*,*N*′,*N*′-tetramethyl-*p*-phenylenediamine.

## Introduction

**Scheme 1:**
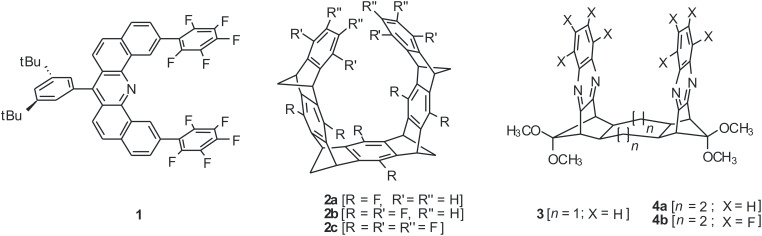
Fluorinated molecular tweezers.

A broad variety of structurally diverse molecular tweezers, i.e., scaffolds in which a tether unit connects two *syn*-oriented aromatic pincers, are well-established as devices for the molecular recognition of mostly electron-deficient guest compounds [1–10]. Conversely, molecular tweezers with a binding cleft that displays an inverted electrostatic potential could thus find application in sensing of electron-rich guests, or even anions [11–13]. Possible frameworks include the seemingly trivial fluorinated analogues of known frameworks (Scheme 1), but so far only a few groups have investigated these intriguing target compounds: Korenaga and Sakai optimized the synthetic access to fluorinated acridine-based molecular tweezers **1** and determined association constants for the complexation of electron-rich arenes [14–15]. Hermida-Ramón and Estévez calculated the structures and electrostatic potentials of belt-shaped compounds **2a**–**c** and predicted the complexation of halide anions in the cavity of **2c** [16–18].

Intrigued by Chou’s communication on the spectroscopic properties of non-fluorinated bis-quinoxalines of type **3** and **4a** [19], we targeted on the corresponding fluorinated derivatives – in particular compound **4b** with its large binding cleft.

In this paper, we present the synthesis and characterization of these synthetically more challenging derivatives. Furthermore, we discuss structural features of a cyclooctadiene-derived scaffold of type **4b** and report preliminary spectroscopic data on their association with electron-rich guest compounds.

## Results and Discussion

### Synthesis of fluorinated bis-quinoxalines

**Scheme 2:**
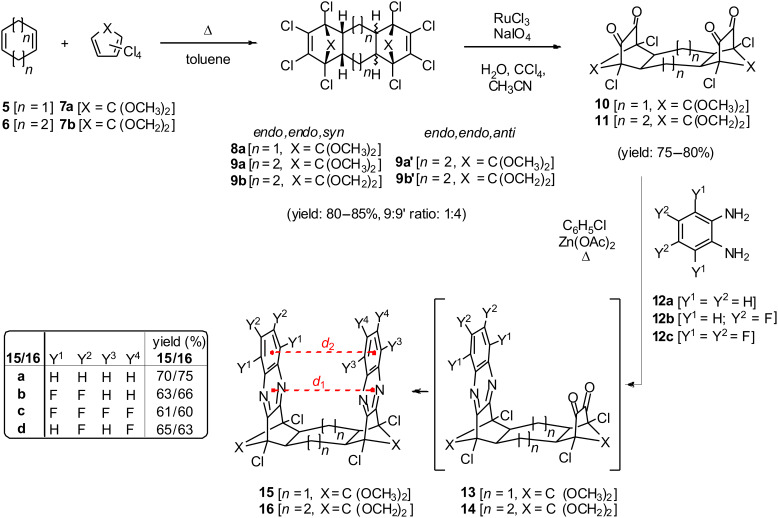
Synthesis of non-fluorinated and fluorinated *syn*-bis-quinoxalines.

The general route [19] to bis-quinoxaline targets (Scheme 2) utilizes a twofold Diels–Alder reaction of a cycloalkadiene (**5**,**6**) with cyclopentadienone derivatives (**7**), subsequent oxidation of the *syn*-diene intermediates (**8**,**9**) to their corresponding tetraketones (**10**,**11**) and condensation of the latter with *o*-phenylenediamine derivatives (**12**) to obtain the *syn*-bis-quinoxaline target compounds (**15**,**16**). This synthetic route is flexible with regard to the tether size (cyclohexane vs cyclooctane) and modifications in the pincer sidewalls (degree of fluorination).

Although only the larger cyclooctadiene-derived scaffolds **16a**–**d** could function as molecular tweezers, we also synthesized the fluorinated cyclohexadiene-derived compounds **15b**–**d** with their smaller π-π–distances. A Diels–Alder reaction of cyclohexadiene (**5**) with ketal **7a** furnished exclusively the *syn*-bis-adduct **8a** [20] which was then converted to the canary-yellow tetraketone **10** by Khan’s original RuCl_3_-catalyzed oxidation protocol [21–23] since Chou’s “optimized” procedure was somewhat capricious in our hands. The twofold condensation with di- or tetrafluoro-*o*-phenylenediamine (**12b**,**c**) [24–25] provided access to the novel fluorinated species **15b**–**c** in acceptable yields (60–70%). This last reaction required harsh conditions and delivered a dark crude product with unspecified tarry material after heating the substrates for several days to 115 °C (^1^H and ^19^F NMR control). Occasionally, the condensation reaction did not lead to complete conversion of the tetraketone precursor **10** and produced a separable mixture of the mono- and bis-condensation products **13** and **15**, respectively. The isolated mono-adducts **13a** (or **13b**) could then be converted to the symmetrical target **15a** (or **15c**) or, upon condensation with the appropriate *o*-phenylenediamine derivative **12c** (or **12a**), to scaffold **15b** with only one fluorinated quinoxaline subunit.

The synthetic access to cyclooctadiene-derived scaffolds is complicated by the lack of selectivity in the twofold Diels–Alder reaction of diene **6** and led to a mixture of the *syn*- and *anti*-bis-adducts in a 1:4 ratio [26–27]. Since the separation of the crucial *syn*-isomer **9a** from *anti*-compound **9a′** by repeated recrystallization did not furnish the pure *endo*,*endo*,*syn*-isomer **9a** in our hands, we focused on the new spirocyclic derivative **9b**. Thus, reaction of the spiro-ketal **7b** [28] with cyclooctadiene (**6**) furnished a mixture of **9b** and **9b′** in excellent yield in the same ratio of isomers as observed in the previous case. Again, the *endo*,*endo*,*syn*-isomer **9b** could not be satisfactorily separated from the *endo*,*endo*,*anti*-isomer **9b′** by chromatography, but gram-amounts of the crucial *syn*-isomer **9b** were readily obtained after repeated recrystallization from hot diethyl ether. The assignment of both *syn*- and *anti*-isomers was initially based on ^1^H NMR spectroscopic analogies to the bis-methoxyketals, i.e., the small low-frequency shift of the bridgehead proton resonances of the *anti*-adduct (Δδ = 0.20 ppm). The X-ray structure determination of target compound **16c** confirmed indirectly the correct assignment of isomers **9b** and **9b′** (vide infra). Oxidation of **9b** with Khan’s original protocol [21–23] and condensation of the resulting tetraketone **11** with *o*-phenylenediamine **12a** or the fluorinated derivatives **12b**–**c** resulted in the new non-fluorinated parent compound **16a** and the three fluorinated scaffolds **16b**–**c**, respectively. All new *syn*-bis-quinoxalines were purified by flash-chromatography on silica gel and obtained as off-white powders in 60–75% yield after recrystallization from methanol. Considering the low nucleophilicity of the fluorinated amine building blocks **12b**–**c**, our yields in the condensation reaction are quite good (71–86% for each condensation step) and any modification of the reaction conditions by other reported procedures [29–32] did not significantly alter the outcome. It should be noted that all fluorinated bis-quinoxalines are stable compounds which do not show any decomposition over extended periods of time; loss of fluorine has only been observed under typical nucleophilic aromatic substitution conditions.

Although the new compounds, in particular the cyclohexadiene-derived species **15b**–**c**, were reasonably soluble in dipolar aprotic solvents (DMSO, DMF) or halogenated aromatic solvents (C_6_H_5_Cl), they only displayed poor solubility in several standard organic solvents (CHCl_3_, CH_2_Cl_2_, CH_3_OH, C_6_H_6_, CH_3_CN). Their full characterization and some preliminary host–guest studies of the cyclooctadiene-derived frameworks could however, be carried out in dilute chloroform, acetonitrile and methylene chloride solutions. The spectroscopic characteristics of **15a** and several non-fluorinated derivatives have been described elsewhere [19] and the NMR spectroscopic data for **15b**–**c** (**16a**–**d**) are only altered by the absence of the corresponding proton resonances, the additional coupling of fluorine with either the arene protons in **15c** (**16c**) or the aromatic carbon atoms, and the more complex signal structure of the spirocyclic ketal in **16a**–**d**. The UV–vis spectra (available in the Supporting Information File 1) display the expected electronic transitions for quinoxaline derivatives [33–35], i.e., a prominent π,π* transition with λ_max_ between 236–245 nm and a lower intensity *n*,π* transition with λ_max_ between 312–316 nm with a poorly resolved vibrational structure. The spectra of the cyclohexadiene-derived scaffolds **15** and the cyclooctadiene-derived frameworks **16** are very similar. Within each series we could not observe a gradual blue-shift for the electronic transitions as the degree of fluorination increased from **15a** (**16a**) to **15c** (**16c**), a result that is in accord with Chou’s UV–vis data for differently substituted bis-quinoxaline scaffolds that abstain from clear trends as the electronic-withdrawing character of the substituents were altered [19]. The ESI-mass spectra (acetonitrile, acetic acid) of all new *syn*-bis-quinoxalines show the correct isotopic pattern of the protonated molecules and, interestingly, display mass clusters for the protonated “dimers” of compounds **15b** and **16b**. Nevertheless, any interpretation of the nature of these latter species (proton-bridged “dimer”, protonated π-π–aggregate, protonated self-associated “dimer”) requires further investigation and cannot be easily transferred to the solution- or solid-state structures of the neutral tweezer compounds [36].

### Structures

**Figure 1:**
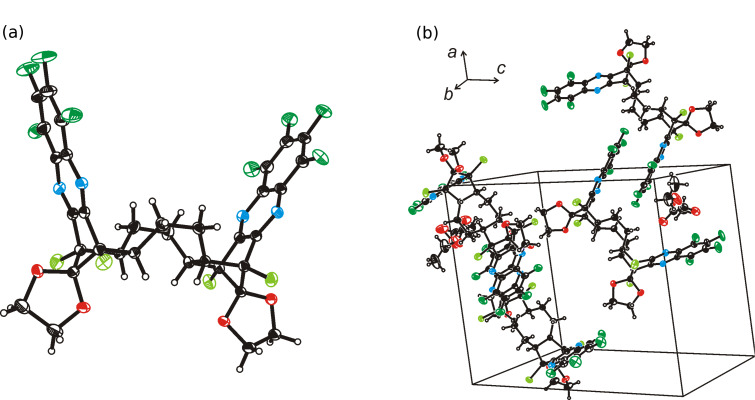
(a) Thermal ellipsoid image of the tweezer molecular **16c** in the structure **16c** · CH_3_CO_2_C_2_H_5_; thermal ellipsoids are drawn at the 50% probability level. (b) View of the packing of **16c** in the unit cell (two CH_3_CO_2_C_2_H_5_ molecules are omitted for clarity). [C_34_H_20_Cl_4_F_8_N_4_O_4_ · CH_3_CO_2_C_2_H_5_, MW = 930.44, monoclinic, space group *P*2_1_/*n*, *a* = 15.3990(12) Å, *b* = 14.0635(11) Å, *c* = 17.7148(14) Å, β = 94.6470(10)°, *V* = 3823.8(5) Å^3^, *Z* = 4, *d*_calc_ = 1.616 g cm^−3^, *T* = 153(2) K, λ = 0.71073 Å, 44247 reflections measured, 9378 unique (*R*_int_ = 0.017), final *R*_1_ [*I* > 2δ*(I)*] = 0.0343 and *R*_1_ = 0.0403 (*wR*_2_ = 0.0952) for all data; CCDC deposit # 786086.

We were able to grow single crystals of the octafluoro compound **16c** from acetonitrile or chloroform solutions suitable for X-ray structure determination (Table 1, Figure 1). In each case, the crystals contained residual ethyl acetate from the purification step, indicating strong binding of the ethyl acetate molecule inside the binding cleft of **16c**. Compound **16c** crystallizes, with an ethyl acetate solvent molecule, in the monoclinic system (space group: *P*2_1_/*n*) and displays bond lengths and angles in the expected ranges. The ethyl acetate displays a small degree of orientation disorder (11.8%). Figure 1a shows a thermal ellipsoid image of **16c** and Figure 1b depicts the packing within a unit cell setting. The large binding pocket of *syn*-bis-quinoxaline **16c** provides enough space to allow the association with solvent (ethyl acetate) and, through “dimer formation”, with the pincer sidewall of a second tweezer molecule. The “dimer” association of fluorinated molecular tweezers in the solid state has been observed for the acridine-derived scaffold **1** [15] and is quite common in many other molecular tweezer scaffolds [1–10]. Compound **16c** shows the typical orientation of fluorine substituents of one pincer sidewall over the arene subunit of another tweezer (substituent distances to arene plane: 3.283 Å, 3.315 Å), interpreted as the attractive interaction between fluorine substituents with the electron-depleted fluoroarene subunit [37–38]. The centroid-centroid distances (*d*_1_, *d*_2_) and the bite angle between the two quinoxaline sidewalls of the binding pocket in fluorinated framework **16c** differ only slightly from the parameters of the non-fluorinated compound **4a**, although the latter does not include any solvent in the cleft and, furthermore, lacks the interpenetrating self-association displayed in **16c** [19]. Conversely, **4a** shows π-π–interaction of two adjacent molecules by stacking two pincer sidewalls, each from the outside (U···U geometry).

**Table 1:** Crystallographic details for **16c** and related non-fluorinated compounds.

	**16c**	**4a** [19]	**3** [19]

crystal system	monoclinic	monoclinic	orthorhombic
space group	*P*2_1_/*n*	*P*2_1_/*c*	*Pbcn*
R [%]	3.79	3.16	3.70
d_1_ [Å]^a^	8.144	7.907	4.686
d_2_ [Å]^a^	10.004	9.641	4.135
bite angle [°]^b^	46.68	45.03	−14.41

^a^defined in Scheme 2. ^b^a negative bite angle defines U- vs. V-shaped tweezers.

### Host–Guest Chemistry

**Figure 2:**
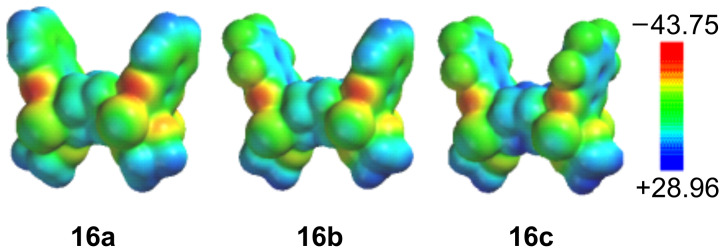
Electrostatic potential surfaces of **16a–c** (Spartan 06 [41]: B3LYP/6-31G*//B3LYP/6-31G*; legend in kcal/mol).

Although none of the reported cyclooctadiene-derived *syn*-bis-quinoxaline scaffolds [19] has been established as a molecular tweezer, the general architecture with two *syn*-oriented aromatic sidewalls and a large π-π–distance does allow the accommodation of guest compounds as demonstrated in the crystal structure of **16c**. Whilst most molecular tweezers have a typical cleft size of ca. 7 Å, several functional larger systems have been reported [39–40]. Figure 2 shows the electrostatic potential surfaces of compounds **16a**–**c**, depicting the inversion of the electrostatic potential in the pincer subunits upon increasing the degree of fluorination.

**Figure 3:**
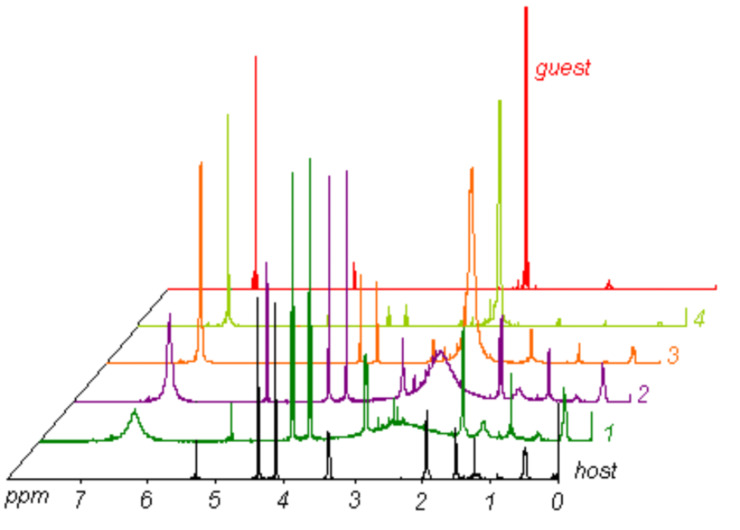
^1^H NMR spectra (CD_2_Cl_2_, 500 MHz) of **16c** (host [black]) upon titration with *N*,*N*,*N*′,*N*′-tetramethyl-*p*-phenylenediamine (guest [red]); host concentration: 0.01M; host–guest ratio: 1:2 (1), 1:5 (2), 1:10 (3), 1:20 (4).

NMR titration experiments with electron-rich arenes (1,4-dimethoxybenzene, 1,3,5-trimethoxybenzene, *N*,*N*-dimethylaniline, *N*,*N*,*N*′,*N*′-tetramethyl-*p*-phenylenediamine) were carried out in deuterated methylene chloride solution for the four cyclooctadiene-derived species **16a**–**d**. Interestingly, only the octafluoro-derivative **16c** showed line-broadening of the ^1^H resonances for one guest compound, i.e., *N*,*N*,*N*′,*N*′-tetramethyl-*p*-phenylenediamine, at various host–guest ratios (Figure 3). No changes in chemical shift of the quinoxaline ^19^F resonances were observed in the ^19^F NMR spectra. Upon cooling the NMR samples the guest’s aromatic and methyl ^1^H resonances sharpened only to less broad signals. Titration of **16c** with other electron-rich aromatic guest compounds (1,4-dimethoxybenzene, 1,3,5-trimethoxybenzene, *N*,*N*-dimethylaniline) under the same conditions showed only the original host and guest resonances in the ^1^H NMR spectra without any line broadening, which indicates that there was no interaction between these three molecules with the tweezer’s cavity. It is important to note that from the entire series of compounds, only the highly fluorinated scaffold **16c** shows chemical exchange between the unbound and bound guest, *N*,*N*,*N*′,*N*′-tetramethyl-*p*-phenylenediamine. While this facile exchange is certainly due to the large binding cleft, the effect of eight fluorine substituents on the electrostatic potential within the cleft is paramount in the facilitation of this interaction between host and guest.

Korenaga and Sakai have already noted that *N*,*N*,*N*′,*N*′-tetramethyl-*p*-phenylendiamine displays a stronger association constant with molecular tweezer **1** when compared to several other electron-rich aromatic guest compounds. This behavior was explained by the large magnitude of the former guest’s quadrupole moment [15].

With our preliminary NMR titrations we could demonstrate that scaffold **16** can indeed associate with an external guest compound in solution if the host and guest units are matched appropriately. Further experiments employing complementary analytical techniques, e.g., isothermal calorimetry, as well as additional investigations of the host–guest chemistry with suitable, larger guest compounds, will provide detailed thermodynamic parameters of the host–guest association, and possibly a better host–guest match, respectively.

## Conclusion

The synthesis of fluorinated *syn*-bis-quinoxalines (**15b**–**c**, **16b**–**c**) was successfully accomplished by a three-step procedure, utilizing the new, readily isolable spirocyclic *syn*-derivative **9b** as an entry towards the larger cyclooctadiene-derived scaffold **16**. The crystal structure of **16c** clearly demonstrates that *syn*-bis-quinoxaline frameworks can function as molecular tweezers. Furthermore, preliminary NMR spectroscopic titration experiments with the octafluoro-*syn*-bis-quinoxaline **16c** prove the interaction of an external, electron-rich guest with the molecular tweezer’s cavity in solution.

## Supporting Information

File 1 Experimental details and characterization data for all new compounds.

File 2 Crystallographic data of *syn*-bis-quinoxaline **16c**.
