# Supplementary material for: Preparation, structures and preliminary host–guest studies of fluorinated syn-bis-quinoxaline molecular tweezers
Source: Beilstein J Org Chem. 2010 Apr 20;6:39. doi: 10.3762/bjoc.6.39 (PMC2874330; doi:10.3762/bjoc.6.39)

Supporting Information File 1

Experimental details and characterization for all new compounds

Preparation, structures and host–guest chemistry of fluorinated *syn*-bis-quinoxaline molecular tweezers

Markus Etzkorn*[1], Jacob C. Timmerman[1], Matthew D. Brooker[1], Xin Yu[2] and Michael Gerken[2]

Address: [1]Department of Chemistry, The University of North Carolina at Charlotte, 9201 University City Blvd., Charlotte, NC 28223, USA and [2]Department of Chemistry and Biochemistry, University of Lethbridge, Lethbridge, AB T1K 3M4, Canada

Email: Markus Etzkorn* - metzkorn@uncc.edu

*Corresponding author

1. Instruments and materials

Melting points are uncorrected and were determined in unsealed capillary tubes on a MEL-TEMP® Electrothermal apparatus. IR spectra were recorded as KBr pellets on a Thermo-Matteson Satellite 3000 FTIR spectrophotometer. UV–vis spectra were recorded on a Varian Cary 300 Bio UV–vis spectrophotometer. Mass spectra, using electrospray ionization were obtained on a PerSpective Biosystems: Mariner Biospectrometry Workstation model, using Mariner Instrument Control Panel v. 4.0.0.0 software and Data Explorer v. 4.0.0.1 software for data analysis. NMR spectra were measured on a 300 MHz Joel 300 ECX or 500 MHz Joel ECA 500 instrument. All spectra were recorded at 298 K and the chemical shifts are reported in ppm, assigning tetramethylsilane [δ(TMS) = 0.00 ppm] as the reference. Signal assignments are based on homo- and heteronuclear 2D NMR experiments and on comparison with analogous

compounds when applicable. Signals indicated with *, **, … are mutually interchangeable. The signal structure is indicated with the usual abbreviations for singlet (s), doublet (d), … Multiplets are quoted over the entire signal range or as centered multiplet. If a multiplet has an apparent triplet structure it is quoted as t_{app}. Elemental analysis was carried out by Atlantic Microlab, Inc.

All starting materials were used as purchased or prepared from commercially available precursors as referenced in the main text of the paper. Solvents were dried by the usual standard procedures.

2. Preparative procedures and characterization of products

Diels-Alder reaction of cyclooctadiene (6) with ketal (7a): The reaction was carried out as reported by Garcia, J. G.; Fronczek, F. R.; McLaughlin, M. L. *Tetrahedron Lett.* **1991**, *32*, 3289–3292. Nevertheless, we were only able to obtain up to 80% enriched *syn*-bis-adduct **9a**.

Diels-Alder reaction of cyclooctadiene (6) with spiroketal (7b): A mixture of cyclooctadiene **6** (3 g, 29 mmol) and spiroketal **7b** (15 g, 57 mmol) was refluxed in toluene (5 ml) for three hours. The beige paste was filtered, washed with methylene chloride (70 ml), dried and washed again with methanol (ca. 15 ml) to remove small amounts of the mono-Diels–Alder adduct. The remaining colorless solid (14.5 g, 83%) contained a 1:4 mixture of **9b** (R_f = 0.43; cyclohexane/ethyl acetate: 4/1) and **9b'** (R_f = 0.41 [cyclohexane/ethyl acetate: 4/1), respectively. After repeated recrystallization from hot diethyl ether the pure *syn*-isomer **9b** was obtained.

***syn*-1',6',7',8',9',14',15',16'-Octachloro-dispiro[1,3-dioxolane-2,17'-pentacyclo[12.2.1.1^{6,9}.0^{2,13}.0^{5,10}]octadecane-18',2''-[1,3]dioxolane]-7',15'-diene (9b):** mp = 286 °C (decomposition);

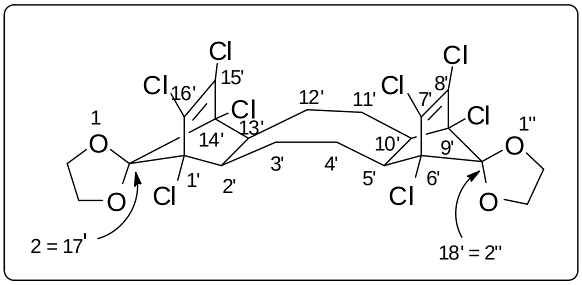

IR (KBr): $\tilde{\nu}$ = 2948, 2908 (CH_2), 1596 (C=C), 1475 (CH_2 deformation), 1249, 1221, 1179, 1151, 1106, 1035, 1003, 940, 885, 856, 810, 769, 730 cm^{-1}; ^1H NMR ($CDCl_3$, 300 MHz): δ = 4.23–4.10 (m, 8H; H-4, -5, -4'', -5''), 2.95–2.82 (m, 4H; H-2', -5', -10', -13'), 2.01–1.87 (m, 4H; H-3', -4', -11', -12') , 0.95–0.77 (m, 4H; H-3', -4', -11', -12'); ^{13}C NMR ($CDCl_3$, 75.6 MHz): δ = 128.7 (C-7', -8', -15', -16'), 120.7 (C-17', -18'), 77.9 (C-1', -6', -9', -14'), 67.6* (C-4, -4''), 66.4* (C-5, -5''), 46.5 (C-2', -5', -10', -13'), 19.8 (C-3', -4', -11', -12'); EA ($C_{22}H_{20}Cl_8O_4$): calc. C (41.81), H (3.19); found C (41.76), H (3.21).

***anti*-1’,6’,7’,8’,9’,14’,15’,16’-Octachloro-dispiro[1,3-dioxolane-2,17’-pentacyclo[12.2.1.1^{6,9}.0^{2,13}.0^{5,10}]octadecane-18’,2’’-[1,3]dioxolane]-7’,15’-diene (9b’):** mp > 295 °C (decomposition); IR (KBr): $\tilde{\nu}$ = 2952, 2905 (CH_2), 1596 (C=C), 1467 (CH_2 deformation), 1355, 1284, 1267, 1245, 1222, 1181, 1132, 1105, 1091, 1037, 1009, 946, 891, 851, 809, 770, 730 cm^{-1}; ^{1}H NMR ($CDCl_3$, 300 MHz): δ = 4.20–4.10 (m, 8H; H-4, -5, -4’’, -5’’), 2.78-2.62 (m, 4H; H-2’, -5’, -10’, -13’), 2.20–2.00 (m, 4H; H-3’, -4’, -11’, -12’), 0.95–0.75 (m, 4H; H-3’, -4’, -11’, -12’); ^{13}C NMR ($CDCl_3$, 75.6 MHz): δ = 128.5 (C-7’, -8’, -15’, -16’), 120.5 (C-17’, -18’), 77.6 (C-1’, -6’, -9’, -14’), 67.7* (C-4, -4’’), 66.5* (C-5, -5’’), 51.8 (C-2’, -5’, -10’, -13’), 21.9 (C-3’, -4’, -11’, -12’); EA ($C_{22}H_{20}Cl_8O_4$): calc. C (41.81), H (3.19); found C (41.78), H (3.16).

$RuCl_3$-catalyzed oxidation of *syn*-diene 8a: The method was analogous to the procedure outlined below (R_f (**10**) = 0.15; cyclohexane/ethyl acetate: 4/1).

$RuCl_3$-catalyzed oxidation of *syn*-diene 9b: A suspension of sodium periodate (1.4 g, 6.5 mmol) and diene **9b** (1 g, 1.6 mmol) was stirred in a mixture of carbon tetrachloride (9 ml), acetonitrile (9 ml) and water (2 ml) at 0 °C. The addition of $RuCl_3 \cdot 3H_2O$ (50 mg) led to an immediate color change to orange-yellow. The reaction was vigorously stirred at 0 °C for several hours and subsequently at room temperature for several days. Once the organic phase turned canary-yellow, water (20 ml) was added. The organic phase was separated, washed with brine, dried ($MgSO_4$) and concentrated *in vacuo* to yield the crude product **11** (R_f = 0.11; cyclohexane/ethyl acetate: 4/1). After triturating in hexanes the bright yellow product (680 mg, 78%) was obtained.

***syn*-1’,6’,9’,14’-Tetrachloro-dispiro[1,3-dioxolane-2,17’-pentacyclo[12.2.1.1^{6,9}.0^{2,13}.0^{5,10}] octadecane-18’,2’’-[1,3]dioxolane]-7’,8’,15’,16’-tetrone (11):** mp > 320 °C; IR (KBr): $\tilde{\nu}$ = 2925, 2910 (CH_2), 1770, 1761 (C=O), 1475, 1252, 1223, 1127, 1091, 1033, 1091, 996, 952, 898, 885, 831, 788, 774, 758, 731 cm^{-1}; ^{1}H NMR ($CDCl_3$, 300 MHz): δ = 4.40–4.23 (m, 8H; H-4, -5, -4’’, -5’’), 3.12–2.99 (m, 4H; H-2’, -5’, -10’, -13’), 2.98–2.95 (m, 4H; H-3’, -4’, -11’, -12’), 1.35–1.24 (m, 4H; H-3’, -4’, -11’, -12’); ^{13}C NMR ($CDCl_3$, 75.6 MHz): δ = 187.9 (C-7’, -8’, -15’, 16’), 110.8 (C-17’, -18’), 78.9 (C-1’, 6’, -9’, -14’), 68.0* (C-4, -4’’), 67.4* (C-5, -5’’), 42.3 (C-2’, -6’, -9’, -14’), 20.5 (C-3’, -4’, -11’, -12’); MS(ESI): *m/z* = 552.99 (M+H^{+}; (correct isotopic pattern), *m/z*

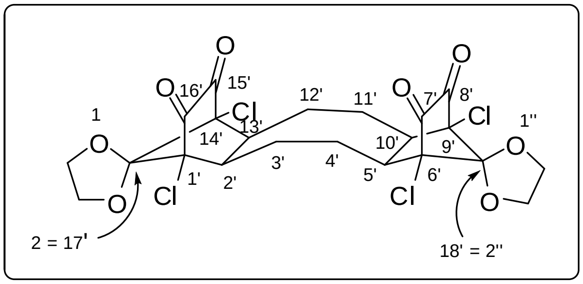

= 589.38 ([M·$2H_2O$+H]$^+$; correct isotopic pattern); EA ($C_{22}H_{20}Cl_4O_8$): calc. C (47.68), H (3.64); found C (47.76), H (3.61).

Condensation of tetraketone intermediates (10, 11) with *ortho*-phenylenediamine derivatives (12): A suspension of the tetraketone (200 mg), *o*-phenylenediamine (2.2 eq.) and zinc acetate (25 mg) was heated in chlorobenzene (2 ml) in an ampoule flask at 115 °C for several days. Once the reaction was complete or no further conversion was observed (NMR control) the dark-brown mixture was cooled to room temperature. After adding water (10 ml) the suspension was sonicated for 10 min and the organic material extracted with chloroform. The dried ($MgSO_4$) organic phase was concentrated *in vacuo* to yield the crude product mixture. After flash-chromatography on a Biotage Isolera system (KP-Sil, 25g cartridge, cyclohexane:ethyl acetate [8:1], $\lambda_{detection}$ = 254 nm and 280 nm) the *syn*-bis-quinoxaline was eluted as the main fraction. The *syn*-bis-quinoxalines were isolated as off-white solids that can be further purified by trituration (methanol) or recrystallization from acetonitrile. The isolated yields for each *syn*-bis-quinoxaline are given below, prior to the analytical data.

R_f (quinoxaline **13**) ≈ 0.17; cyclohexane/ethyl acetate: 4/1

R_f (*syn*-bis-quinoxaline **15**) ≈ 0.22; cyclohexane/ethyl acetate: 4/1

R_f (quinoxaline **14**) ≈ 0.15; cyclohexane/ethyl acetate: 4/1

R_f (*syn*-bis-quinoxaline **16**) ≈ 0.19; cyclohexane/ethyl acetate: 4/1

<u>MONO-QUINOXALINES:</u>

1,5,8,12-Tetrachloro-22,23,24,25-tetramethoxyheptacyclo[10.10.1.1^{5,8}.0^{2,11}.0^{4,9}.0^{13,22}.0^{15,20}]tetracosane-6,7-dione (13b, Y^1=Y^2=F): ^{1}H NMR ($CDCl_3$, 500 MHz): δ = 3.74 (s, 3H; OCH_3), 3.65 (s, 3H; OCH_3), 3.43 (s, 3H; OCH_3), 3.32 (s, 3H; OCH_3), 3.04–2.88* (m, 2H; H-2, -11), 2.79–2.66 (m, 2H; H-4, -9), 2.01–1.91 (m, 2H; H_{anti}-3, -10), -0.36-(-0.52) (m, 2H; H_{syn}-3, -10) ; ^{19}F NMR ($CDCl_3$, 470.6 MHz): δ = -149.8 (m, 2F), -151.9 (m, 2F); ^{13}C NMR ($CDCl_3$, 125.7 MHz): δ = 187.3 (C-6, -7), 153.6 (C-13,-22), 141.8* (dm, C-16, -19), 141.5* (dm, C-17,-18), 129.1 (C-15, -20), 78.9** (C-1, -12), 74.4** (C-5, -8), 52.7 (OCH_3), 52.6

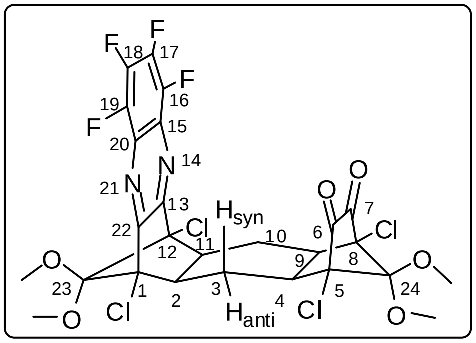

(OCH_3), 52.3 (OCH_3), 52.1 (OCH_3), 43.6*** (C-2, -11), 41.8*** (C-4, -9), 19.0 (C-3, -10); $^1J_{CF}$ = 257 Hz.

1’,6’,9’,14’-Tetrachloro-18’,19’,20’,21’-tetrafluoro-dispiro[1,3-dioxolane-2,25’-[16,23]diaza-heptacyclo[12.10.1.1^{6,9}.0^{2,13}.0^{5,10}.0^{15,24}.0^{17,22}]hexacosa-15’,17’(22’),18’,20’,23’-pentaene-26’,2’’-[1,3] dioxolane]-7’,8’-dione (14b, Y^1=Y^2=F): ^{1}H NMR ($CDCl_3$, 500 MHz): δ = 4.50–4.10 (set of 4m, 8H; H-4, -5, -4’’, -5’’), 3.32-3.22* (m, 2H; H-2’, -13’), 3.21-3.11* (m, 2H; H-5’, -10’), 2.08-1.98** (m, 2H; H_{anti}-3’, -12’), 1.90–1.80** (m, 2H; H_{anti}-4’, -11’), 1.14-1.01*** (m, 2H; H_{syn}-3’, -12’), 0.73-0.60 (m, 2H; H_{syn}-3’, -12’); ^{19}F NMR ($CDCl_3$, 470.6 MHz): δ = -151.1 (m, 2F), -152.8 (m, 2F); ^{13}C NMR ($CDCl_3$, 125.7 MHz): δ = 188.0 (C-7’, -8’), 155.6 (C-15’, -24’), 141.5* (dm, C-18’, -21’), 141.2 (dm, C-19’, -20’), 129.3 (C-17’, -22’), 79.1** (C-1’, 14’), 74.1 (C-6’, -9’), 68.1*** (C-4), 68.0*** (C-5), 67.9*** (C-4’’), 67.8*** (C-5’’), 44.1$^{\#}$ (C-2’, -13’), 42.3$^{\#}$ (C-5’, -10’), 20.7$^{\#\#}$ (C-3’, -12’), 20.3$^{\#}$ (C-4’, -11’) ; $^1J_{CF}$ = 259 Hz.

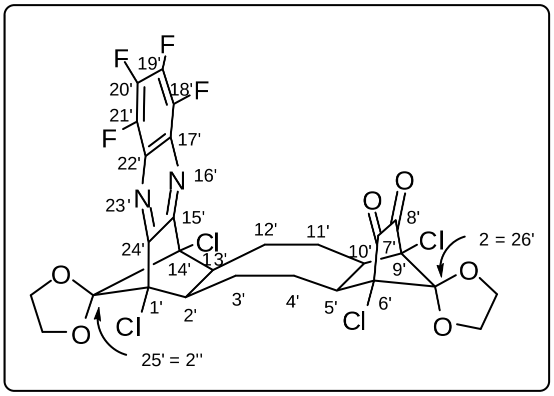

<u>***SYN*-BIS-QUINOXALINES (15b-d)**</u>

1,5,16,20-Tetrachloro-9,10,11,12-tetrafluoro-31,31,32,32-tetramethoxy-7,14,22,29-tetraaza-nonacyclo[18.10.1.1^{5,16}.0^{2,19}.0^{4,17}.0^{6,15}.0^{8,13}.0^{21,30}.0^{23,28}]dotriaconta-6,8(13),9,11,14,21,23(28),24,26,29-decaene (15b): yield: 63%; mp > 320 °C; UV–vis (CH_3CN): λ_{max} (ε) = 239 (51000), 315 (11000) nm ($mol^{-1}dm^3cm^{-1}$); IR (KBr): $\tilde{\nu}$ = 2952, 2845, 1662, 1508, 1491, 1464, 1349, 1307, 1187, 1103, 1035, 1001, 957, 934, 912, 885, 817, 802, 783, 734 cm^{-1}; ^{1}H NMR ($CDCl_3$, 500 MHz): δ = 7.95–7.85* (m, 2H; H-24, -27), 7.72–7.62* (m, 2H, H-25, -26), 3.75 (s, 3H, OCH_3), 3.73 (s, 3H, OCH_3), 3.36 (s, 3H, OCH_3), 3.35 (s, 3H, OCH_3), 2.96–2.80 (m, 4H; H-2, -4, -17, -19), 2.15–1.85 (m, 2H; H_{anti}-3, -18), -1.15-(-1.40) (m, 2H; H_{syn}-3, -18); ^{19}F NMR ($CDCl_3$, 470.6 MHz): δ = -150.2 (m, 2F), -152.4 (m, 2F); ^{13}C NMR ($CDCl_3$, 125.7 MHz): δ = 153.6* (C-6, -15), 152.2* (C-21, -30), 141.0** (dm, C-9, -12), 139.8** (dm, C-10, -11), 129.3*** (C-24, -27), 128.8*** (C-25, -

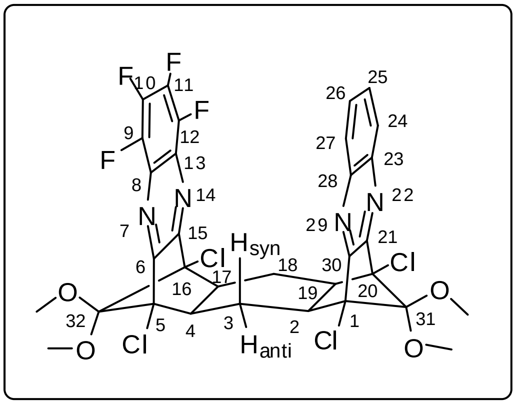

26), 128.7 (C-8, -13, -23, -28), 111.2**** (C-32), 111.1**** (C-31), 74.7# (C-5, -16), 74.6# (C-1, -20), 42.9## (C-4, -17), 42.8## (C-2, -19), 19.7 (C-3, -18); ${}^{1}J_{CF}$ = 260 Hz; MS(ESI): *m/z* = 745.15 ($[M+H]^+$; correct isotopic pattern), 1489.30 ($[2M+H]^+$; correct isotopic pattern); EA ($C_{32}H_{24}Cl_4F_4N_4O_4$): calc. C (51.5), H (3.24); found C (51.37), H (3.19).

1,5,16,20-Tetrachloro-9,10,11,12,24,25,26,27-octafluoro-31,31,32,32-tetramethoxy-7,14,22,29-tetraazanonacyclo[18.10.1.1^{5,16}.0^{2,19}.0^{4,17}.0^{6,15}.0^{8,13}.0^{21,30}.0^{23,28}]dotriaconta-6,8(13),9,11,14,21,23(28),24,26,29-decaene (15c): yield: 61%; mp > 320 °C; UV–vis (CH_3CN): λ_{max} (ε) = 241 (87000), 312 (12000) nm ($mol^{-1}dm^3 cm^{-1}$); IR (KBr): $\tilde{\nu}$ = 2952, 2871, 1665, 1523, 1494, 1465, 1344, 1300, 1206, 1184, 1158, 1109, 1080, 1041, 992, 969, 933, 885, 821, 794, 731, 697 cm^{-1}; ^{1}H NMR ($CDCl_3$, 500 MHz): δ =3.72 (s, 6H, OCH_3), 3.38 (s, 6H; OCH_3), 2.98–2.82 (m, 4H; H-2, -4, -17, -19), 2.05–1.94 (m, 4H; H_{anti}-3, -18), -1.22-(-1.42) (m, 4H; H_{syn}-3, -18); ^{19}F NMR ($CDCl_3$, 470.6 MHz): δ = -149.8 (m, 4F), -152.4 (m, 4F); ^{13}C NMR ($CDCl_3$, 125.7 MHz): δ = 154.1 (C-6, -15, -21, -30), 141.2* (dm, C-9, -12, -24, -27), 141.1* (dm, C-10, -11, -25, -26), 128.3 (C-8, -13, -23, -28), 111.2 (C-31, -32), 75.8 (C-1, -5, -16, -20), 52.5 (OCH_3), 52.2 (OCH_3), 42.6 (C-2, -4, -17, -19), 19.8 (C-3, -18); ${}^{1}J_{CF}$ = 261 Hz; MS-ESI *m/z* = 817.132 ($[M+H]^+$, correct isotopic pattern); EA ($C_{32}H_{20}Cl_4F_8N_4O_4$): calc. C (46.97), H (2.46); found C (47.04), H (2.38).

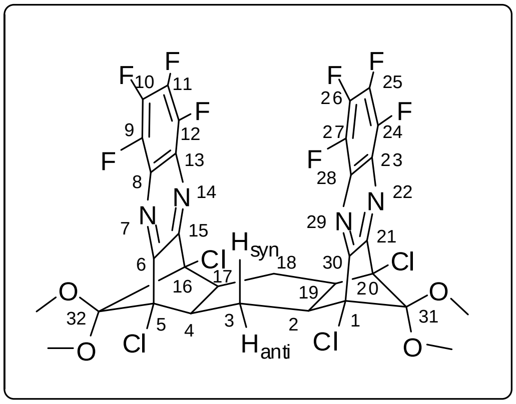

1,5,16,20-Tetrachloro-10,11,25,26-tetrafluoro-31,31,32,32-tetramethoxy-7,14,22,29-tetra-azanonacyclo[18.10.1.1^{5,16}.0^{2,19}.0^{4,17}.0^{6,15}.0^{8,13}.0^{21,30}.0^{23,28}]dotriaconta-6,8(13),9,11,14,21, 23(28),24,26,29-decaene (15d): yield: 65%; mp. > 320 °C; UV–vis (CH_3CN): λ_{max} (ε) = 236 (26000), 315 (12000) nm ($mol^{-1}dm^3cm^{-1}$); IR (KBr): $\tilde{\nu}$ = 2918, 2850, 1644, 1519, 1466, 1442, 1378, 1308, 1251, 1214, 1191, 1148, 1105, 1046, 1029, 1002, 970, 948, 881, 864, 813, 764, 751, 693, 669 cm^{-1}; ^{1}H NMR $CDCl_3$, 500 MHz): δ = 7.63–7.52 (t_{app}, 4H; H-9, -12, -27, -24), 3.73 (s, 6H; OCH_3), 3.35 (s, 6H; OCH_3), 3.00–2.72 (m, 4H; H-2, -4, -17, -19); 2.01-1.89 (m,

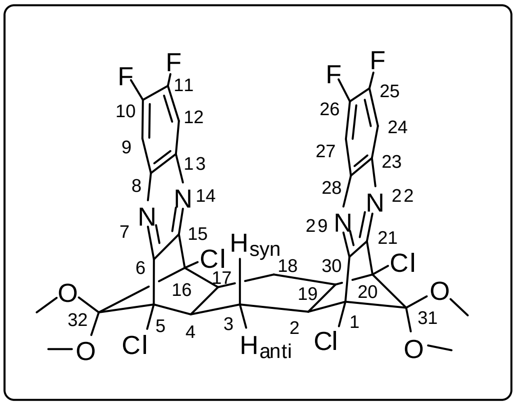

4H; H_{anti}-3, -18), -1.22-(-1.39) (m, 4H; H_{syn}-3, -18); ^{19}F NMR ($CDCl_3$, 470.6 MHz): δ = -129.55 (m); ^{13}C NMR ($CDCl_3$, 125.7 MHz): δ = 152.6 (C-6, -15, -21, -30), 141.8 (dm, C-10, -11, -25, -26), 138.5 (C-9, -12, -24, -27), 114.9 (C-8, -13, -23, -28), 111.3 (C-31, -32), 74.6 (C-1, -5, -16, -20), 52.5 (OCH_3), 52.2 (OCH_3), 43.0 (C-2, -4, -17, -19), 19.7 (C-3, -18); $^1J_{CF}$ = 258 Hz; MS(ESI): *m/z* = 745.1567 ($[M+H]^+$, correct isotopic pattern); EA ($C_{32}H_{24}Cl_4F_4N_4O_4$): calc. C (51.5), H (3.24); found C (51.34), H (3.19).

<u>*SYN*-BIS-QUINOXALINES (16a-d):</u>

1’,6’,17’,22’-Tetrachloro-dispiro[1,3-dioxolane-2,33’-[8’,15’,24’,31’]-tetraazanonacyclo [20.10.1.1^{6,17}.0^{2,21}.0^{5,18}.0^{7,16}.0^{9,14}.0^{23,32}.0^{25,30}]tetratriacontane-34’,2’’-[1,3]dioxolane]-7,9(14),10,12,15,23,25(30),26,28,31-decaene (16a): yield: 75% mp > 320 °C; UV–vis (CH_3CN): λ_{max} (ε) = 241 (69000), 319 (17000) nm ($mol^{-1}dm^3cm^{-1}$); IR (KBr): $\tilde{\nu}$ = 2896, 1625, 1591 1560, 1508, 1472, 1397, 1373, 1321, 1245, 1222, 1142, 1121, 1094, 1036, 1017, 961, 901, 875, 834, 806, 766, 658 cm^{-1}; ^1H NMR ($CDCl_3$, 500 MHz): δ = 8.17–8.09* (m, 4H; H-10’, =13’, -26’, -29’), 7.75–7.68* (m, 4H; H-11’, -12’, -27’, -28’), 4.42** (t_{app}, 4H; H-4, -4’’), 4.20** (t_{app}, 4H; H-5, -5’’), 3.41–3.3.29 (m, 4H; H-2’, -5’, -18’, -21’), 2.01–1.92 (m, 4H, H-3’, -4’, -19’, -20’), 0.62–0.43 (m, 4H; H-3’, -4’, -19’, -20’); ^{13}C NMR ($CDCl_3$, 125.7 MHz): δ = 152.1 (C-7’, -16’, -23’, 32’), 142.4 (C-9’, -14’, -25’, -30’), 129.9* (C-11’, -12’, -27’, -28’), 129.3* (C-10’, -13’, -26’, -29’), 120.1 (C-33’, 34’), 74.4 (C-1’, -6’, -17’, -22’), 67.9** (C-5, -5’’), 67.1** (C-4, -4’’), 44.4 (C-2’, -5’, -18’, -21’), 20.5 (C-3’, -4’, -19’, -20’); MS(ESI): *m/z* = 697.22 ($[M+H]^+$, correct isotopic pattern); EA ($C_{34}H_{28}Cl_4N_4O_4$): calc. C (58.47), H (4.84); found C (58.62), H (4.90).

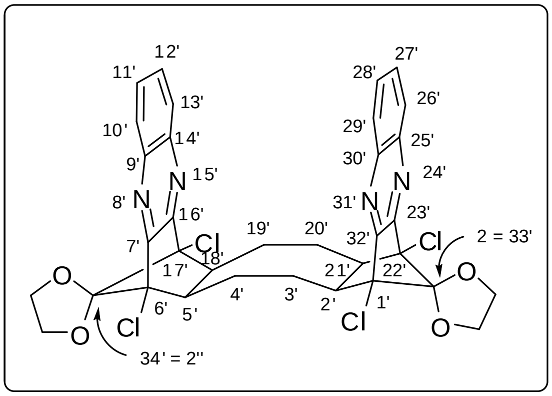

1’,6’,17’,22’-Tetrachloro-10’,11’,12’,13’-tetrafluoro-dispiro[1,3-dioxolane-2,33’-[8’,15’,24’, 31’]-tetraazanonacyclo[20.10.1.1^{6,17}.0^{2,21}.0^{5,18}.0^{7,16}.0^{9,14}.0^{23,32}.0^{25,30}]tetratriacontane-34’,2’’-[1,3]dioxolane]-7,9(14),10,12,15,23,25(30),26,28,31-decaene (16b): yield: 66%; mp > 320 °C;

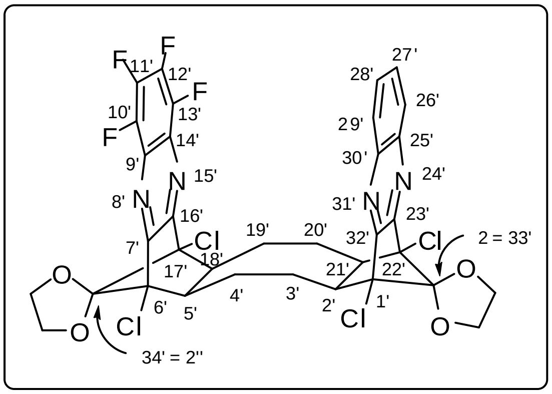

UV–vis (CH_3CN): λ_{max} (ε) = 241 (64000), 315 (13000) nm ($mol^{-1}dm^3cm^{-1}$); IR (KBr): $\tilde{\nu}$ = 2921, 2853, 1663, 1507, 1489, 1376, 1348, 1317, 1221, 1201, 1147, 1121, 1096, 1058, 1037, 997, 971, 956, 880, 833, 806, 766 cm^{-1}; ^{1}H NMR ($CDCl_3$, 500 MHz): δ = 8.18–8.06* (m, 2H; H-26’, -29’), 7.78–7.66* (m, 2H; H-27’, 28’) 4.44** (t_{app}, 4H; H-4, -4’’), 4.19** (m_c, 4H; H-5, -5’’), 3.41-3.21 (m, 4H; H-2’, -5’, -18’, -21’), 2.08–1.89 (m, 4H; H-3’, -4’, -19’, -20’), 0.62–0.35 (m, 4H; H-3’, -4’, -19’, -20’); ^{19}F NMR ($CDCl_3$, 470.6 MHz): δ = −151.3 (m, 2F), −153.2 (m, 2F); ^{13}C NMR ($CDCl_3$, 125.7 MHz): δ = 154.0* (C-7’, -16’), 152.1* (C-23’, -32’), 142.5 (C-25, -30), 141.7** (dm, C-10’, -13’), 141.2** (dm, C-11’, -12’), 130.2*** (C-26’, -29’), 129.6*** (C-27’, -28’), 129.5 (m, C-9’, -14’), 120.1(C-33’, -34’), 74.6**** (C-1’, -22’), 74.7**** (C-6’, -17’), 68.3$^{\#}$ (C-4) 68.1$^{\#}$ (C -4’’), 67.3$^{\#}$ (C-5), 67.2$^{\#}$ (C-5’’), 44.4 (C-2’, -5’, -18’, -21’), 20.7$^{\#\#}$ (C-3’, -20’), 20.6$^{\#\#}$ (C-4’, -19’); $^1J_{CF}$ = 258 Hz; MS(ESI): *m/z* = 769.20 ($[M+H]^+$, correct isotopic pattern); EA ($C_{34}H_{24}Cl_4F_4N_4O_4$): calc. C (53.01), H (3.14); found C (52.97), H (3.18).

1’,6’,17’,22’-Tetrachloro-10’,11’,12’,13’,26’,27’,28’29’-octafluoro-dispiro[1,3-dioxolane-2,33’-[8’,15’,24’,31’]-tetraazanonacyclo[20.10.1.1^{6,17}.0^{2,21}.0^{5,18}.0^{7,16}.0^{9,14}.0^{23,32}.0^{25,30}]tetratria-contane-34’,2’’-[1,3]dioxolane]-7,9(14),10,12,15,23,25(30),26,28,31-decaene (16c): yield:

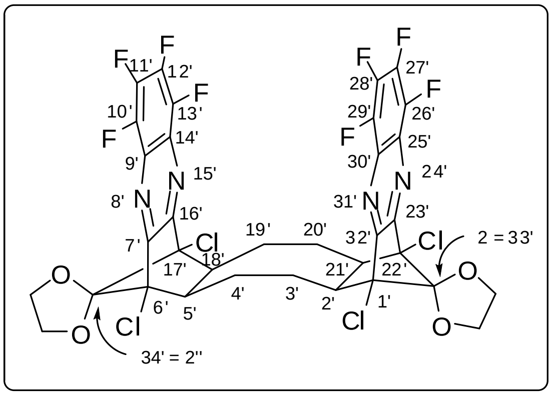

60%; mp > 320 °C; UV–vis (CH_3CN): λ_{max} (ε) = 245 (76000), 316 (11000) nm ($mol^{-1}dm^3cm^{-1}$); IR (KBr): $\tilde{\nu}$ = 2972, 2956, 1662, 1507, 1491, 1347, 1315, 1225, 1203, 1149, 1098, 1036, 1006, 954, 883, 785, 770 cm^{-1}; ^{1}H NMR ($CDCl_3$, 500 MHz): δ = 4.44* (t_{app}, 4H; H-4, -4’’), 4.16* (t_{app}, 4H; H-5, -5’’), 3.41-3.25 (m, 4H; H-2’, -5’, -18’, -21’), 2.05-1.88 (m, 4H; H-3’, -4’, -19’, -20’), 0.62-0.56 (m, 4H; H-3’, -4’, -19’, -20’); ^{19}F NMR ($CDCl_3$, 470.6 MHz): δ = -150.0 (m, 4F), −152.6 (m, 4F); ^{13}C NMR ($CDCl_3$, 125.7

MHz): δ = 153.9 (C-7', -16', -23', -32'), 141.9* (dm, C-10', -13', 26', -29'), 140.2* (dm, C-11', -12', -27', -28'), 129.3 (m, C-9', -14', -25', 30'), 120.1 (C-33', -34'), 74.4 (C-1', -6', -17', -22'), 68.2** (C-4, -4''), 67.3** (C-5, -5''), 44.4 (C-2', -5', -18', -21'), 20.7 (C-3', -4', -19', -20'); $^1J_{CF}$ = 256 Hz; MS(ESI): *m/z* = 841.187 ($[M+H]^+$, correct isotopic pattern); EA($C_{34}H_{20}Cl_4F_8N_4O_4$): calc. C (48.48), H (2.39); found C (48.12), H (2.41).

1',6',17',22'-Tetrachloro-11',12',27',28'-tetrafluoro-dispiro[1,3-dioxolane-2,33'-[8',15',24', 31']-tetraazanonacyclo[20.10.1.1^{6,17}.0^{2,21}.0^{5,18}.0^{7,16}.0^{9,14}.0^{23,32}.0^{25,30}]tetrariacontane-34',2''-[1,3]dioxolane]-7,9(14),10,12,15,23,25(30),26,28,31-decaene (16d): yield: 63%; mp. > 320 °C;

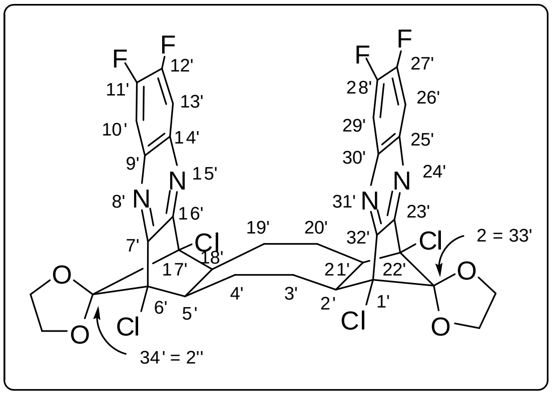

UV–vis (CH_3CN): λ_{max} (ε) = 236 (67000), 316 (31000) nm ($mol^{-1}dm^3cm^{-1}$); IR (KBr): $\tilde{\nu}$ = 2923, 2854, 1515, 1469, 1442, 1265, 1205, 147, 1098, 1039, 1014, 968, 959, 879, 846, 811, 756, 689 cm^{-1}; ^{1}H NMR ($CDCl_3$, 500 MHz): δ = 7.87 (t_{app}, 4H; H-10', -13', -26', -29'), 4.50-4.40* (t_{app}, 4H; H-4, -4''), 4.22-4.12* (t_{app}, 4H; H-5, -5''), 3.37–3.22 (m, 4H; H-2', -5', -18', -21'), 1.98–1.88 (m, 4H; H-3', -4', -19', -20'), 0.68–0.40 (m, 4H; H-3', -4', -19', -20'); ^{19}F NMR ($CDCl_3$, 470.6 MHz): δ = −129.7 (t_{app}, 4F); ^{13}C NMR ($CDCl_3$, 125.7 MHz): δ = 153.9 (C-7', -16', -23', -32'), 141.7 (dm, C-11', -12', -27', -28'), 140.2 (C-10', -13', -26', -29'), 129.2 (m, C-9', -14', -25', 30'), 120.0 (C-33', -34'), 74.3 (C-1', -6', -17', 22'), 68.2* (C-4, -4''), 67.2* (C-5, -5''), 44.4 (C-2', -5', -18', -21'), 20.5 (C-3', -4', -19', -20'); $^1J_{CF}$ = 257 Hz; MS(ESI): *m/z* = 769.20 ($[M+H]^+$, correct isotopic pattern); *EA* *($C_{34}H_{24}Cl_4F_4N_4O_4$):* calc. C (53.01) H (3.14); found C (52.91) H (3.08).

UV–vis spectra of cyclohexadiene-derived *syn*-bis-quinoxalines 15 (c = 5 ×10^{-6} M, CH_3CN):

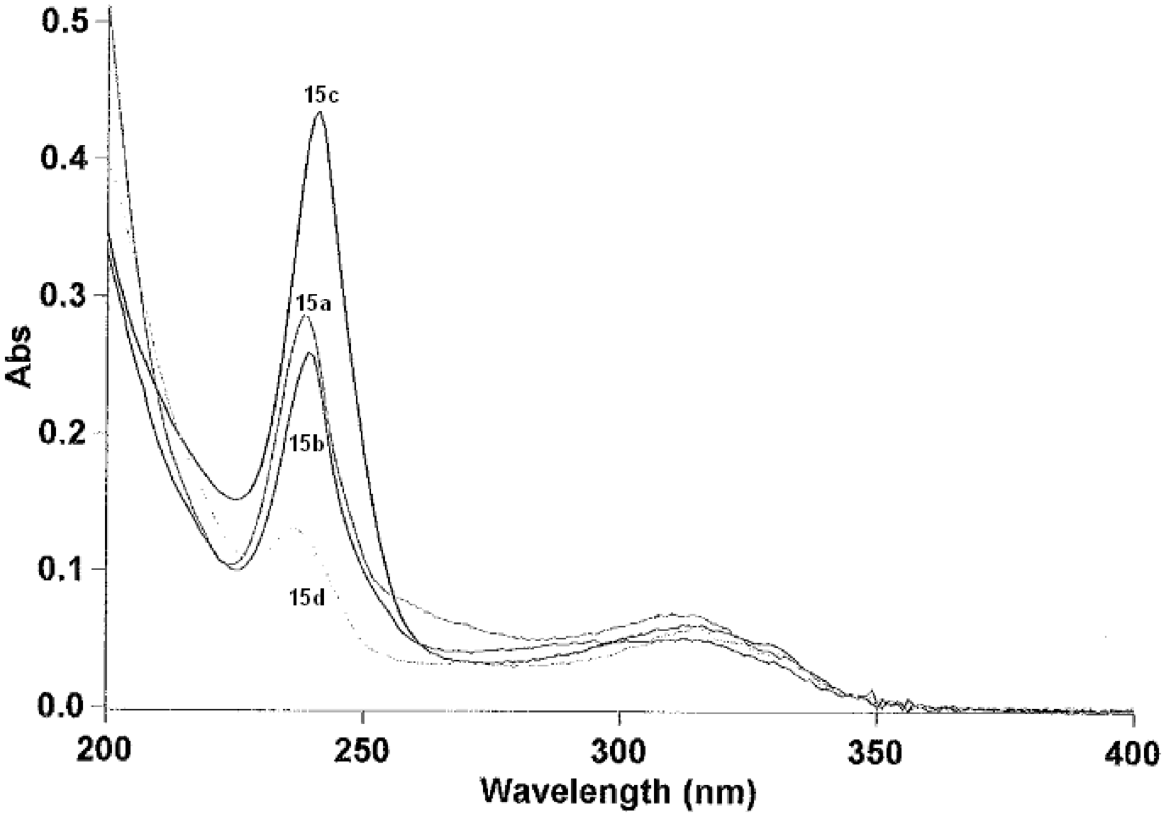

UV–vis spectra of cyclooctadiene-derived *syn*-bisquinoxalines 16 (c = 5 × 10^{-6} M, CH_3CN):

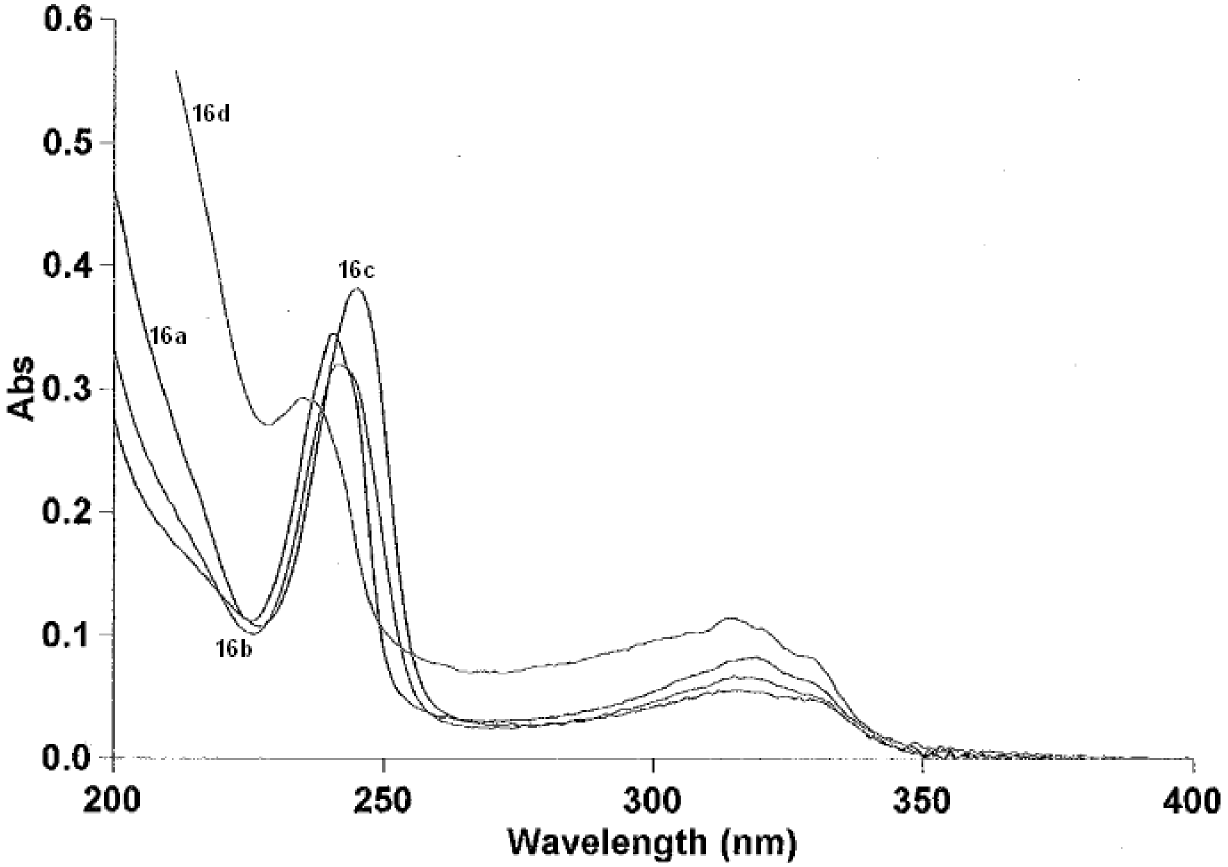

Supplement: File 1 — Experimental details and characterization data for all new compounds. [file Beilstein_J_Org_Chem-06-39-s001.pdf]
