# Supplementary material for: Preparation, structures and preliminary host–guest studies of fluorinated syn-bis-quinoxaline molecular tweezers
Source: Beilstein J Org Chem. 2010 Apr 20;6:39. doi: 10.3762/bjoc.6.39 (PMC2874330; doi:10.3762/bjoc.6.39)
Supplement: File 2 — Crystallographic data of syn-bis-quinoxaline 16c. [file Beilstein_J_Org_Chem-06-39-s002.pdf]

Supporting Information File 2

Crystallographic data of *syn*-bis-quinoxaline, 16c·$CH_3CO_2C_2H_5$;

Preparation, structures and host–guest chemistry of fluorinated *syn*-bis-quinoxaline molecular tweezers

Markus Etzkorn*[1], Jacob C. Timmerman[1], Matthew D. Brooker[1], Xin Yu[2] and Michael Gerken[2]

Address: [1]Department of Chemistry, The University of North Carolina at Charlotte, 9201 University City Blvd., Charlotte, NC 28223, USA and [2]Department of Chemistry and Biochemistry, University of Lethbridge, Lethbridge, AB T1K 3M4, Canada

Email: Markus Etzkorn* - metzkorn@uncc.edu

*Corresponding author

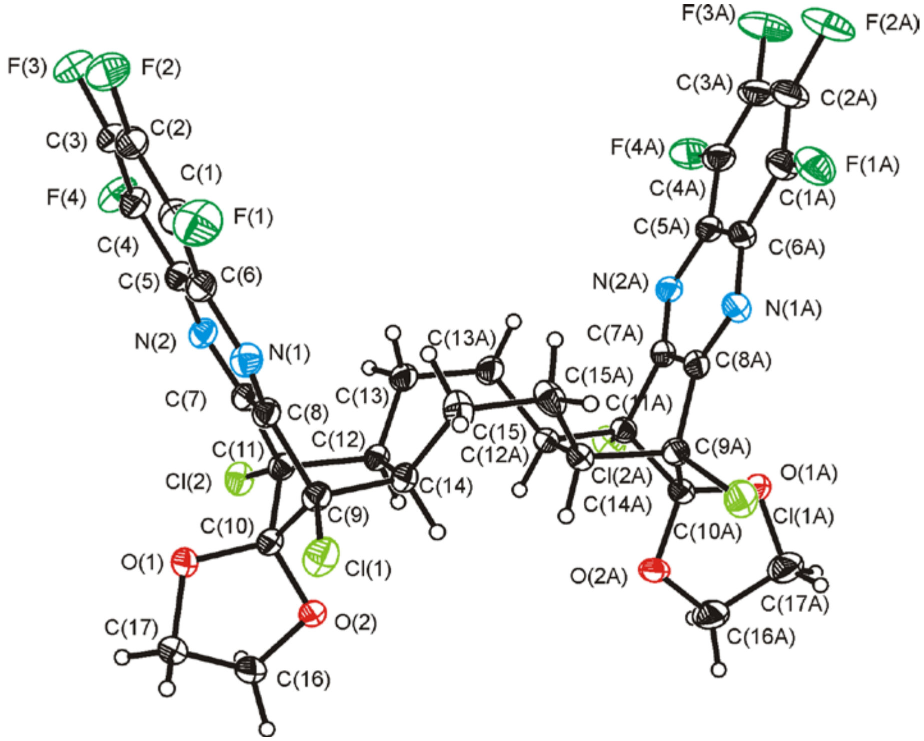

Figure 1: Thermal ellipsoid plot of the *syn*-bis-quinoxaline molecule of **16c**·$CH_3CO_2C_2H_5$; thermal ellipsoids are drawn at the 50% probability level.

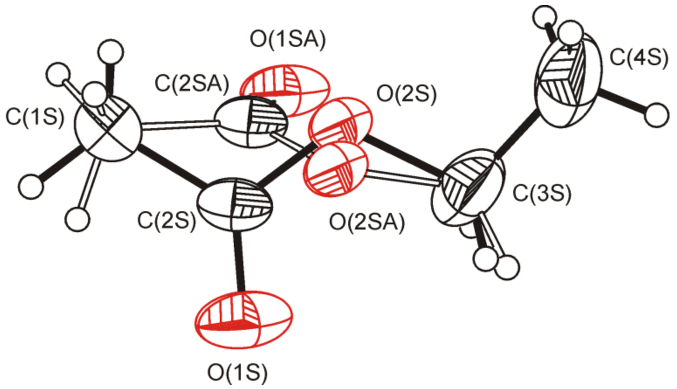

Figure 2: Thermal ellipsoid plot of the disordered ethyl acetate molecule of **16c**·$CH_3CO_2C_2H_5$; thermal ellipsoids are drawn at the 50% probability level.

Table 1: Crystal data and structure refinement for **16c**·$CH_3CO_2C_2H_5$; (IUPAC notation of **16c**: 1',6',17',22'-Tetrachloro-10',11',12',13',26',27',28',29'-octafluoro-dispiro[1.3-dioxolane-2,33'-[9',15',24',31']-tetraazanonacyclo[$20.10.1.^{16,17}.0^{2},^{21}.0^{5,18}.0^{7,16}.0^{9,14}.0^{23,32}.0^{25,30}$]tetratriaconta-7,9(14),10,12,15,23,25(30),26,28,31-decaene-34',2''-[1,3]dioxolane]).

Empirical formula	$C_{38}H_{28}Cl_2F_8N_4O_6$	
Formula weight	930.44	
Temperature, K	153(2)	
Wavelength, Å	0.71073	
Crystal system	monoclinic	
Space group	$P2_1/n$	
Unit cell dimensions	a = 15.3990(12) Å	α = 90º
	b = 14.0635(11) Å	β = 94.6470(10)º
	c = 17.7148(14) Å	γ = 90º
Volume, $Å^3$	3823.8(5)	
Z	4	
Density (calculated), g cm^{-1}	1.616	
Absorption coefficient, mm^{-1}	0.403	
F(000)	1888	
Crystal size	0.53 x 0.43 x 0.29 mm^3	
theta range for data collection	1.69 to 28.84º	
Limiting indices	$-20 \le h \le 20$, $-19 \le k \le 19$, $-23 \le l \le 24$	
Reflections collected	44247	
Independent reflections	9378 (R_{int} = 0.017)	
Refinement method	Full-matrix least-squares on F^2	
Data / restraints / parameters	9378 / 4 / 554	
Goodness-of-fit on F^2	1.032	
Final R indices [I>2sigma(I)]	R_1 = 0.0343, wR_2 = 0.0903	
R indices (all data)	R_1 = 0.0403, wR_2 = 0.0952	
Extinction coefficient	0	
Largest diff. peak and hole, $eÅ^{-3}$	0.41 and -0.37	

Table 2: Geometric Parameters (Ǻ, º).

C1—F1	1.3338(19)	C1A—F1A	1.343(2)
C1—C2	1.369(2)	C1A—C2A	1.364(2)
C1—C6	1.415(2)	C1A—C6A	1.411(2)
C2—F2	1.3391(17)	C2A—F2A	1.3425(19)
C2—C3	1.396(3)	C2A—C3A	1.396(3)
C3—F3	1.3415(18)	C3A—F3A	1.3420(19)
C3—C4	1.368(2)	C3A—C4A	1.368(2)
C4—F4	1.3372(19)	C4A—F4A	1.341(2)
C4—C5	1.410(2)	C4A—C5A	1.410(2)
C5—N2	1.3794(18)	C5A—N2A	1.3775(19)
C5—C6	1.421(2)	C5A—C6A	1.423(2)
C6—N1	1.3829(19)	C6A—N1A	1.3779(19)
N1—C7	1.3026(18)	N1A—C7A	1.2988(18)
N2—C8	1.2995(18)	N2A—C8A	1.3012(18)
C7—C8	1.4375(19)	C7A—C8A	1.4353(19)
C7—C11	1.5068(18)	C7A—C11A	1.5057(19)
C8—C9	1.5038(18)	C8A—C9A	1.5070(18)
C9—C14	1.5624(18)	C9A—C10A	1.5590(19)
C9—C10	1.5647(19)	C9A—C14A	1.5626(18)
C9—Cl1	1.7592(13)	C9A—Cl1A	1.7602(14)
C10—O2	1.3933(16)	C10A—O2A	1.3971(16)
C10—O1	1.3985(16)	C10A—O1A	1.3980(16)
C10—C11	1.5586(19)	C10A—C11A	1.561(2)
C11—C12	1.5632(18)	C11A—C12A	1.5618(19)
C11—Cl2	1.7595(14)	C11A—Cl2A	1.7595(14)
C12—C13	1.5328(19)	C12A—C13A	1.5304(19)
C12—C14	1.5892(18)	C12A—C14A	1.5849(18)
C14—C15	1.5250(18)	C14A—C15A	1.5286(19)
O1—C16	1.4403(17)	O1A—C16A	1.4328(19)

O2—C17	1.4441(18)	O2A—C17A	1.4415(19)
C16—C17	1.512(2)	C16A—C17A	1.498(3)
C13—C13A	1.527(2)	C15—C15A	1.529(2)
C1S—C2S	1.490(3)		
C2S—O1S	1.195(3)	C2SA—O1SA	1.187(15)
C2S—O2S	1.346(3)	C2SA—O2SA	1.325(16)
O2S—C3S	1.447(3)		
C3S—C4S	1.489(4)		
C1S—C2S	1.490(3)		

F1—C1—C2	119.60(14)	F1A—C1A—C2A	119.60(14)
F1—C1—C6	119.95(14)	F1A—C1A—C6A	119.66(14)
C2—C1—C6	120.44(15)	C2A—C1A—C6A	120.73(16)
F2—C2—C1	120.81(16)	F2A—C2A—C1A	120.36(18)
F2—C2—C3	118.57(15)	F2A—C2A—C3A	119.10(16)
C1—C2—C3	120.61(14)	C1A—C2A—C3A	120.53(15)
F3—C3—C4	120.30(16)	F3A—C3A—C4A	120.47(18)
F3—C3—C2	119.10(14)	F3A—C3A—C2A	119.02(16)
C4—C3—C2	120.58(14)	C4A—C3A—C2A	120.50(15)
F4—C4—C3	119.26(14)	F4A—C4A—C3A	119.86(14)
F4—C4—C5	120.23(13)	F4A—C4A—C5A	119.61(14)
C3—C4—C5	120.46(15)	C3A—C4A—C5A	120.52(16)
N2—C5—C4	118.46(13)	N2A—C5A—C4A	118.65(14)
N2—C5—C6	122.35(13)	N2A—C5A—C6A	122.38(13)
C4—C5—C6	119.17(13)	C4A—C5A—C6A	118.97(14)
N1—C6—C1	118.91(14)	N1A—C6A—C1A	118.83(14)
N1—C6—C5	122.37(12)	N1A—C6A—C5A	122.45(13)
C1—C6—C5	118.72(14)	C1A—C6A—C5A	118.71(14)
C7—N1—C6	113.27(12)	C7A—N1A—C6A	113.32(13)
C8—N2—C5	113.57(12)	C8A—N2A—C5A	113.26(12)

N1—C7—C8	124.26(13)	N1A—C7A—C8A	124.25(13)
N1—C7—C11	129.53(13)	N1A—C7A—C11A	129.48(13)
C8—C7—C11	106.18(11)	C8A—C7A—C11A	106.26(11)
N2—C8—C7	124.11(12)	N2A—C8A—C7A	124.30(13)
N2—C8—C9	129.59(12)	N2A—C8A—C9A	129.66(13)
C7—C8—C9	106.29(11)	C7A—C8A—C9A	106.04(11)
C8—C9—C14	108.57(10)	C8A—C9A—C10A	99.67(11)
C8—C9—C10	99.82(10)	C8A—C9A—C14A	107.98(11)
C14—C9—C10	100.64(10)	C10A—C9A—C14A	101.44(10)
C8—C9—Cl1	115.10(9)	C8A—C9A—Cl1A	115.74(10)
C14—C9—Cl1	114.63(9)	C10A—C9A—Cl1A	115.71(10)
C10—C9—Cl1	116.21(9)	C14A—C9A—Cl1A	114.43(9)
O2—C10—O1	109.39(11)	O2A—C10A—O1A	109.10(11)
O2—C10—C11	111.38(11)	O2A—C10A—C9A	114.27(11)
O1—C10—C11	114.68(11)	O1A—C10A—C9A	112.26(11)
O2—C10—C9	114.11(11)	O2A—C10A—C11A	112.52(11)
O1—C10—C9	112.94(11)	O1A—C10A—C11A	114.59(11)
C11—C10—C9	93.73(10)	C9A—C10A—C11A	93.54(10)
C7—C11—C10	100.15(10)	C7A—C11A—C10A	99.84(11)
C7—C11—C12	108.11(11)	C7A—C11A—C12A	108.11(11)
C10—C11—C12	100.61(10)	C10A—C11A—C12A	101.14(10)
C7—C11—Cl2	115.05(10)	C7A—C11A—Cl2A	115.25(10)
C10—C11—Cl2	115.52(10)	C10A—C11A—Cl2A	115.67(10)
C12—C11—Cl2	115.44(9)	C12A—C11A—Cl2A	114.94(9)
C13—C12—C11	112.23(11)	C13A—C12A—C11A	112.05(11)
C13—C12—C14	117.85(12)	C13A—C12A—C14A	117.29(12)
C11—C12—C14	102.82(10)	C11A—C12A—C14A	102.68(10)
C13A—C13—C12	117.83(12)	C13—C13A—C12A	118.30(12)
C15—C14—C9	112.68(11)	C15A—C14A—C9A	111.40(11)
C15—C14—C12	117.58(11)	C15A—C14A—C12A	118.34(12)

C9—C14—C12	102.69(10)	C9A—C14A—C12A	102.77 (10)
C14—C15—C15A	117.85(11)	C14A—C15A—C15	117.84 (12)
C10—O1—C16	108.05(10)	C10A—O1A—C16A	107.81 (11)
C10—O2—C17	106.61(10)	C10A—O2A—C17A	107.57(11)
O1—C16—C17	103.91(11)	O1A—C16A—C17A	103.06(13)
O2—C17—C16	102.70(11)	O2A—C17A—C16A	103.79(13)
O1S—C2S—O2S	123.3(2)	C2S—O2S—C3S	116.1(2)
O1S—C2S—C1S	125.7(2)	O2S—C3S—C4S	105.3(2)
O2S—C2S—C1S	110.89(18)	O1SA—C2SA—O2SA	121.6(15)

X-ray Structure Determination:

The bulk crystals were colorless. The crystal used for X-ray data collection had the dimensions 0.53 × 0.43 × 0.29 mm^3.

Collection and Reduction of X-ray Data

X-ray diffraction data were collected using a Bruker SMART APEX II diffractometer equipped with an APEX II 4K charge-coupled device (CCD) area detector (by use of the program APEX2) [1] and a sealed-tube X-ray source (graphite-monochromated Mo Kα radiation, λ = 0.71073 Å). A complete sphere of data was collected to better than 0.8 Å resolution. Processing was carried out by using the program SAINT [2], which applied Lorentz and polarization corrections to three-dimensionally integrated diffraction spots. The program SADABS [3] was used for the scaling of diffraction data, the application of a decay correction and an empirical absorption correction based on redundant reflections.

Solution and Refinement of the Structure

All calculations were performed using the SHELXTL Plus package [4] for structure determination, refinement and molecular graphics. The XPREP program [4] was used to confirm the unit cell dimensions and the crystal lattice. A solution was obtained using direct methods. Successive difference Fourier syntheses revealed all atoms. The final refinement was obtained by introducing

a weighting factor and anisotropic thermal parameters for all non-hydrogen atoms. A solvent molecule of ethyl acetate was found in the crystal lattice. Residual electron density around the ethyl acetate indicated positional disorder of the acetate moiety. The disorder was modeled and the site occupancy of the disordered atoms was refined to 88.2 vs. 11.8%.

References

1. Bruker APEX2, Version 2.2-0, Madison, WI, 2007.
2. SAINT+, Version 7.46A, Madison, WI, 2007.
3. Sheldrick, G. M. SADABS, Version 2007/4, Bruker AXS Inc., Madison, WI, 2007.
4. Sheldrick, G. M. SHELXL97, University of Göttingen, Göttingen, 1997.
